# Functional Roles and Host Interactions of *Orthoflavivirus* Non-Structural Proteins During Replication

**DOI:** 10.3390/pathogens14020184

**Published:** 2025-02-12

**Authors:** Meghan K. Donaldson, Levi A. Zanders, Joyce Jose

**Affiliations:** 1 Department of Biochemistry and Molecular Biology, The Pennsylvania State University, University Park, PA 16802, USA; mkd6021@psu.edu (M.K.D.); levizanders@psu.edu (L.A.Z.); 2 The Huck Institutes of the Life Sciences, The Pennsylvania State University, University Park, PA 16802, USA

**Keywords:** flavivirus non-structural proteins, replication complex, assembly, virus-host interactions, tick-borne, mosquito-borne, innate immunity, intracellular movement, interactome, antivirals, vaccines, membrane modification

## Abstract

*Orthoflavivirus*, a genus encompassing arthropod-borne, positive-sense, single-stranded RNA viruses in the *Flaviviridae* family, represents clinically relevant viruses that pose significant threats to human and animal health worldwide. With warming climates and persistent urbanization, arthropod vectors and the viruses they transmit continue to widen their geographic distribution, expanding endemic zones. Flaviviruses such as dengue virus, Zika virus, West Nile virus, and tick-borne encephalitis virus cause debilitating and fatal infections globally. In 2024, the World Health Organization and the Pan American Health Organization declared the current dengue situation a Multi-Country Grade 3 Outbreak, the highest level. FDA-approved treatment options for diseases caused by flaviviruses are limited or non-existent, and vaccines are suboptimal for many flaviviruses. Understanding the molecular characteristics of the flavivirus life cycle, virus-host interactions, and resulting pathogenesis in various cells and model systems is critical for developing effective therapeutic intervention strategies. This review will focus on the virus-host interactions of mosquito- and tick-borne flaviviruses from the virus replication and assembly perspective, emphasizing the interplay between viral non-structural proteins and host pathways that are hijacked for their advantage. Highlighting interaction pathways, including innate immunity, intracellular movement, and membrane modification, emphasizes the need for rigorous and targeted antiviral research and development against these re-emerging viruses.

## 1. Introduction

The family *Flaviviridae* consists of enveloped virions with icosahedral symmetry that contain a positive-sense, single-stranded RNA genome ~11 kilobases (kb) [1]. Four genera are in the family *Flaviviridae*: *Pegivirus*, *Hepacivirus*, *Pestivirus*, and *Orthoflavivirus*. Notable members include hepatitis G and C viruses (*Pegivirus* and *Hepacivirus*, respectively), bovine viral diarrhea virus (BVDV) and classical swine fever virus (CSF) (*Pestivirus*), and dengue virus (DENV), Zika virus (ZIKV), West Nile virus (WNV), yellow fever virus (YFV), and tick-borne encephalitis virus (TBEV) (*Orthoflavivirus*) [1].

Members of the *Orthoflavivirus* genus, hereafter referenced as flaviviruses, are arthropod-borne viruses (arboviruses) transmitted by vectors such as mosquitoes and ticks infecting birds, ruminants, ungulates, and other small mammals, with humans serving as incidental and dead-end hosts. The more human-adapted members, such as DENV, ZIKV, YFV, and TBEV, cause severe, debilitating, or fatal diseases in humans [2]. DENV is rapidly proving to be a serious re-emerging health risk, with worldwide laboratory-confirmed cases surpassing seven million in 2024 alone and deaths approaching 10,000. According to the World Health Organization (WHO), dengue is the most common mosquito-borne disease on Earth [3]. ZIKV is also a re-emerging concern due to mammalian vertical transmission from mother to offspring, particularly in humans [4]. Tick-borne flaviviruses, such as TBEV, Powassan virus (POWV), and Kyasanur forest disease virus (KFDV), remain prominent health concerns due to their encephalitic or hemorrhagic pathogenesis and continued expansion [5,6,7]. Flaviviruses lack treatment options beyond supportive care, and only a few protective vaccines have been approved for use in humans. The licensed vaccine for YFV, YFV-17D, is a live-attenuated vaccine that has been in use since the 1930s [8]. The live-attenuated chimeric tetravalent DENV vaccine, Dengvaxia, will be discontinued in 2026 due to a lack of global demand and contributing to antibody-mediated enhancement (ADE) [9]. Currently, six licensed inactivated vaccines exist for TBEV in the United States, Canada, Russia, China, and multiple European countries, including FSME-Immun/TicoVac/Encepur [10,11], EnceVir, Tick-E-Vac/Klesch-E-Vac, Sen Tai Bao, and TBE vaccine Moscow [12]. The inactivated vaccine for Japanese encephalitis virus (JEV), IXIARO, has been licensed for use in the United States and the European Union since 2009 [13].

Understanding the molecular characteristics of the flavivirus life cycle, virus-host interactions, and pathogenesis in diverse model systems is critical for developing effective therapeutic interventions. To this end, this review focuses on key components of flavivirus replication, highlighting cellular pathways advantageously hijacked by the virus and potential avenues of therapeutic research. Flavivirus replication involves tightly regulated, host-integrated steps enabling efficient infection. This virus cycle disrupts the local cellular microenvironment and triggers global host dysregulation via altered transcription, diverted cellular materials, and host factor manipulation. Deciphering these virus-host interactions demands powerful molecular techniques.

## 2. Molecular Characteristics of Flaviviruses

### 2.1. Flavivirus Structure and Genomic Components

**Figure 1:**
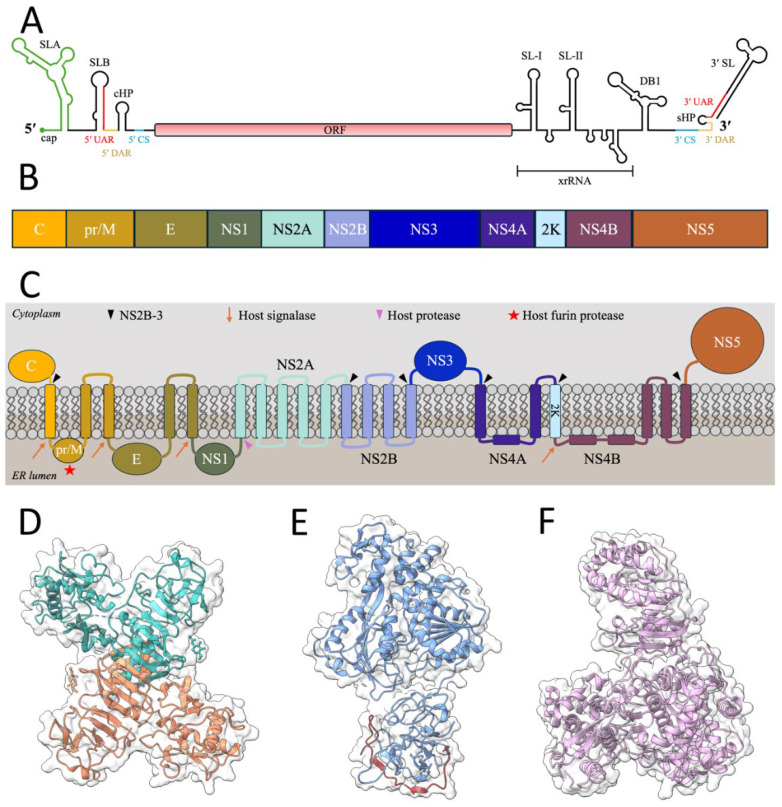
Representation of the flavivirus genome organization and known full-length protein crystal structures. (**A**) ZIKV genomic RNA structure with predicted 5′ and 3′ UTR structures. (**B**) Polyprotein organization. (**C**) Polyprotein topology across the ER membrane with indicated viral and host protease cleavage sites [18]. (**D**) ZIKV NS1 (PDB: 5GS6) dimeric crystal structure solved through X-ray crystallography with each monomer separately colored. (**E**) DENV NS2B-3 (PDB: 5YVW) monomer crystal structure solved through X-ray crystallography with the NS2B peptide colored in red and NS3 in blue. (**F**) ZIKV NS5 (PDB: 5TMH) structure solved through X-ray crystallography.

Flavivirus virions have icosahedral symmetry with a diameter of roughly 50 nm, encapsidating a single, positive-sense RNA genome of about 11 kb that encodes the viral proteins required to replicate and assemble new virions upon infection of naïve cells. The RNA genome is packaged by several copies of the capsid protein (C) within a host-derived lipid bilayer surrounded by an icosahedral shell consisting of 180 copies of both the envelope (E) glycoprotein and membrane (M) protein [14,15]. The smooth icosahedral shell in the mature flavivirus is formed by three dimers of E-M heterodimers (E-M)2 lying parallel to one another, forming a raft structure with a characteristic herringbone pattern [16]. The RNA genome is capped with a type-I cap (m7GpppAmG) during replication, lacks a polyA tail, and contains highly structured 5′ and 3′ untranslated regions (UTRs) flanking a single open reading frame (ORF) (Figure 1A) [17]. Upon translation on the rough endoplasmic reticulum (ER), a single polypeptide is processed into three structural proteins [capsid (C), precursor membrane (prM), envelope (E)], and seven non-structural proteins (NS1, NS2A, NS2B, NS3, NS4A, NS4B, NS5) (Figure 1B) [18]. Flavivirus UTRs are essential for the translation and replication of viral RNA and immune evasion [17]. The secondary structures of the 5′ UTR are important for proper replication and translation, whereas the structures found in the 3′ UTR are essential for immune evasion and host hijacking. The ~100 nucleotide (nt) 5′ UTR contains two secondary domains separated by a uridine-rich linker: a branched stem-loop A structure (SLA) and B structure (SLB) with additional structures downstream (Figure 1A) [19]. SLA is conserved among flaviviruses and serves to recruit the RNA-dependent RNA polymerase (RdRp) for replication. The uridine-rich linker is proposed to enhance viral replication due to the increased flexibility of the RNA molecule, allowing the polymerase to bind to the promoter found in the SLA [19]. The SLB encompasses a region of the capsid ORF (c-ORF) and contains the downstream AUG region (DAR), capsid hairpin structure (cHP), a circularization sequence (CS), and a pseudoknot (PK) (Figure 1A).

The ~400–700 nt 3′ UTR contains three domains: the variable, dumbbell, and conserved regions [20,21]. The variable region contains two stem-loop structures (SL-I and SL-II) that can form pseudoknots (PK1 and PK2, respectively) (Figure 1A). The highly structured region of the 3′ UTR is notably resistant to XRN1, a 5′-3′ exoribonuclease, and thus commonly referred to as XRN1-resistant (xr) RNAs: xrRNA1 or xrRNA2, depending on the region where XRN1 dissociates [22]. In flaviviruses, these xrRNAs also termed sub-genomic flavivirus RNAs (sfRNAs), play essential roles in host innate immune suppression and adaptation to new hosts by hijacking host proteins in both a proviral and antiviral manner [23,24,25]. The dumbbell region varies among flaviviruses as either containing one (ex. ZIKV, YFV: DB1) or two (ex. DENV, JEV: DB1, DB2) dumbbell secondary structures that are conserved and may form pseudoknots (PK3 and PK4, respectively) (Figure 1A) [20,21,26,27]. These DB structures harbor important sequences for replication and translation and may stall XRN1, leading to additional xrRNA species [26]. The conserved domain, containing regions interacting with the 5′ UTR, is important for RNA circularization and viral replication. This domain also includes a short hairpin sequence (sHP) and a terminal stem-loop (SL) that aid RNA synthesis (Figure 1A). Every component of the flavivirus genome is multifunctional and plays essential roles in the replicative cycle.

### 2.2. Flavivirus Life Cycle

Flavivirus entry begins with the E glycoprotein binding to a cell surface receptor or attachment factor. Many attachment factors have been postulated for flaviviruses, including glycosaminoglycans, heat-shock proteins, laminin, and C-type lectins [28]. As flaviviruses infect many cell types, finding a ubiquitous attachment factor has proven challenging [28,29,30]. Following attachment, the virion enters the cell via receptor-mediated endocytosis, likely at or near pre-established clathrin-coated pits on the cell surface [31]. Within the late endosome, low pH triggers conformational changes in the E protein, leading to the exposure of the hydrophobic fusion loop and fusion of the endosomal and viral membranes, generating a fusion pore [31]. This fusion event releases the nucleocapsid into the cytosol, and the C protein dissociates from the viral genomic RNA to be degraded [32]. The genomic RNA is subsequently translated in a cap-dependent manner, co-translationally translocated onto the ER membrane, and processed by both the viral protease (NS2B-NS3) and host proteases co- and post-translationally (Figure 1B,C). NS3 undergoes autocatalytic cleavage and cleaves its cofactor, NS2B, in cis to generate the NS2B-NS3 protease complex (NS2B-3) [33]. When the protease complex is fully formed, viral cytosolic cleavage sites at the C anchor, NS3-NS4A, NS4A-2K, and NS4B-NS5 junctions will be recognized and cut in trans [33]. The small peptide, 2K, is processed from NS4A, serves as a signal sequence for proper NS4B topology, and assists in the functions of NS4A during replication and membrane modification, depending on its cleavage state [34,35]. The viral luminal cleavage sites between the C-prM, prM-E, E-NS1, and 2K-NS4B junctions are recognized by host signal peptidase (SPase) or signalase, with the notable exception of the junction between NS1-NS2A, for which the responsible protease has yet to be elucidated [36,37]. An important cleavage event between the C-prM junction by SPase following viral cleavage results in delayed prM processing, allowing viral replication and nucleocapsid formation [38,39].

Flavivirus RNA replication occurs in replication organelles (ROs), which are invaginations of the ER membrane induced by the non-structural proteins responsible for aiding in the synthesis of new viral genomes [40,41]. The ROs contain the viral RNA replication machinery that utilizes the positive-sense genomic RNA template to synthesize an uncapped negative-sense RNA intermediate. Negative-sense RNA synthesis requires the linear, single-stranded genome to circularize into a panhandle-shaped structure [42]. The negative-sense RNA intermediate is used as a template to synthesize new positive-sense genomes during replication, generating a double-stranded RNA (dsRNA) intermediate that can trigger host innate immune sensors [43]. The dsRNA intermediate is unwound by the viral helicase, NS3, and nascent positive-sense genomes are synthesized and capped by the viral RNA-dependent RNA polymerase (RdRp), NS5 [44]. By asymmetric replication, flaviviruses synthesize multiple copies of the nascent positive-sense genome. This process heavily relies on the *de novo* synthesis of cellular nucleotides and long-range interactions between the viral UTRs [42,45]. The capped nascent viral RNA is presumably extruded from the replication organelle and forms to serve three purposes: used as a new template for replication, translated to form new viral proteins, or packaged to form new virions [46].

Following replication, newly synthesized viral RNAs are transported to assembly sites on the modified ER membrane to be assembled with the C protein into nucleocapsid cores. Although only the newly replicated RNA is packaged into the budding particles, the process by which the RNA transverses the cytosolic space between the RO and the juxtapositioned assembly site remains unsolved [40,47,48,49]. The C protein binds RNA, likely as dimers, to encapsidate genomic RNA and form a nucleocapsid core [50]. It is currently unknown how many C proteins are associated with a nascent genome to form a core and if cores need to be fully formed before packaging. Nucleocapsids bud into regions of the ER membrane embedded with 60 trimeric spikes of prM/E heterodimers, thereby obtaining a lipid bilayer envelope and generating an immature particle. The heterotrimeric spikes alone can drive the budding process, forming non-infectious subviral particles lacking a nucleocapsid core [51]. Nucleocapsids have been found to have an organized structure in the immature virus while rarely being found alone in the cell, indicating that the budding process is very rapid or tightly regulated [52,53,54].

Following budding into the ER lumen, immature particles accumulate in distal, dilated ER cisternae, forming arrays [40,41]. These immature viruses are transported via secretory vesicles along microtubules to the trans-Golgi apparatus. During transport through the Golgi, decreasing pH causes the prM/E spikes to rearrange and collapse, forming a smooth, herringbone-like structure exposing the furin cleavage site on prM (Figure 1A) [55,56]. Host furin protease cleavages the pr peptide from prM, leading to the maturation of the viral particle. The cleaved pr segment is retained on the E protein of the mature particle to prevent premature fusion of the viral envelope with the exocytic vesicle [55,56,57]. The pr peptide is released from the mature virion, preparing the E protein to bind and infect a new cell.

## 3. Structure and Function of Non-Structural Proteins

Note for readers: amino acid (a.a.) numbering for all non-structural proteins hereafter corresponds to the number of residues present in ZIKV proteins unless otherwise described.

### 3.1. Non-Structural Protein 1

Non-structural protein 1 [NS1; 352 a.a. (ZIKV)] is a glycoprotein that exists as a membrane-associated dimer and a soluble hexamer secreted from the infected cell [58]. The oligosaccharyltransferase complex (OST) helps glycosylate NS1, which has a significant role in determining its functional properties, and the position of N-linked glycosylation sites varies among flaviviruses [58,59,60]. TBEV NS1 is known to be glycosylated at Asn23, Asn85, and Asn207, NS1 of DENV, ZIKV, YFV, and JEV are glycosylated at Asn130 and Asn207, and WNV NS1 is glycosylated at Asn130, Asn175, and Asn207 [58,60,61]. The NS1 monomer processed from the viral polyprotein by host proteases readily forms a membrane-associated dimer on the luminal side of the ER membrane or cell surface associated with lipid rafts. NS1 also exists as a soluble hexameric lipoprotein complex when secreted into the extracellular milieu [58].

The structure of NS1 comprises three domains, β-roll, wing, and β-ladder, which have been determined by X-ray crystallography and cryoEM from both the dimer and secreted hexamers of ZIKV, DENV, and WNV (Figure 1D) [60,62,63,64,65]. The N-terminal β-roll is a dimerization domain that interacts with the lipid bilayer in the membrane-associated dimer and forms a hydrophobic interior in the hexamer where a dense lipid core is trapped in the lipoprotein complex. The wing domain is the middle domain of NS1 that contains two subdomains, a disordered loop, and the major conserved N-linked glycosylation sites: Asn130 and Asn175 [60]. It is named accordingly due to its projection from the β-ladder domain with a disulfide bond linker between the two domains. The disordered region of the wing domain forms an external portion of the hexamer lipoprotein readily targeted by the host immune system and is referenced as the spike region. In the membrane-associated form, the spike region and a hydrophobic greasy finger motif enable cellular membrane association [62,63]. The C-terminal β-ladder domain has a core structure containing nine β-chains stacked in a ladder-type appearance with an extending loop between two chains known as the spaghetti loop (Figure 1D) [60]. In the secreted lipoprotein complex, the C-terminal tip of the ladder contains host-recognizable epitopes [60]. A variant of NS1, termed NS1′, has been characterized in WNV, JEV, and DENV arising from a ribosomal frameshift in NS2A, resulting in a 52-amino-acid extension of the C-terminus of the mature protein [66,67,68]. While not confirmed in all flaviviruses, this variant has been shown to have neuroinvasive capabilities and host antiviral regulation properties [67,69]. The functional roles of NS1 are multifaceted within the cell, which makes this protein a compelling target for therapeutic intervention [70,71]. Recently, a soluble tetrameric structure was solved through cryoEM, which the authors postulated to be a more accurate oligomeric representation of the NS1 lipoprotein complex. The predominance and functional significance of this new structure are unclear [64].

NS1 plays a role in replication by associating with NS4A and NS4B on the luminal side of the ER membrane and potentially serves a role in membrane bending to assist in the formation of the RO [72,73]. Amino acid residues within the β-roll dimerization domain (R10, Q11) have been found to interact with NS4B in WNV [74]. Furthermore, hydrophobic residues within the β-roll domain are hypothesized to participate in membrane bending of the RO, albeit to a much smaller extent than NS4A or NS4B [73]. The dipeptide residues (R/N10, Q/K11; WNV/DENV) in the β-roll domain are also postulated to determine the fate of the mature protein in various flaviviruses depending on the strength of the NS1-NS4B interaction: retention in the ER, localization to the plasma membrane, or secretion into the extracellular milieu [72,75]. Additionally, NS1 has known roles in RNA synthesis and associating with structural proteins at assembly sites, potentially affecting nucleocapsid packaging into immature particles [73,75,76]. Notably, within ZIKV, NS1 appears to aid in pathogenesis during vertical transmission, as antibody-mediated neutralization of this protein reduced fetal damage [77]. Strikingly, the β-roll and linker region of the wing domain (1–50 a.a.) of NS1 were shown to induce tunneling nanotubes (TNTs) in placental trophoblasts, allowing for TNT-mediated intercellular transmission of mitochondria and viral proteins between cells and evasion of extracellular sensors [77]. These actin-mediated structures were only previously seen in WNV in Vero E6 cells [78].

NS1 is shown to co-precipitate with many host proteins that are involved in translation, microtubule-based processes, glycolysis, and transport, including the ribosomal protein RPL-18, components of the ER-associated degradation (ERAD)/unfolded protein response (UPR) system like HERPUD1, and the intermediate filament vimentin (Table 1) [79,80]. RPL-18 was shown to localize to the perinuclear region of the infected cell with NS1, the area associated with replication and assembly, and was not dependent upon the presence of viral RNA [79]. Vimentin is an essential intermediate filament for flaviviruses as it is depolarized and reassembled in the perinuclear region to anchor the ROs within the ER [80]. Actin was also shown to participate in RO biogenesis and SUN2-mediated NS1-actin interactions in ZIKV, DENV, and JEV [81].

NS1 induces tissue damage and vascular leakage by affecting the immune system and evading innate immune pathways such as the complement system and TLR/RLR (Toll-like receptor/RIG-I-like receptor) recognition (Table 1) [65,82]. However, the primary role NS1 plays in antagonizing or inhibiting TLR-induced transcriptional activation of IFN-β and NF-κB from TLR2-, TLR6-, and TLR3-mediated pathways has not been well-established among different flaviviruses [83,84]. NS1 is known to target RIG-I, MAVS, CDK1, MDA5, and TBK1 in the RLR pathway; is involved in the production of type-I interferons (IFNs); and inhibits downstream signaling pathways either directly or indirectly [85,86,87,88]. Regarding the complement system, ectopically expressed NS1 has been shown to bind to C4b [89,90]. Validated host factor interactions with NS1 are shown below (Table 1).

**Table 1:** Summary of validated replication- and assembly-related NS1 protein interactors.

Virus-Adapted Function	Host Factor	Flavivirus	Validation Method	Reference
Innate immunity antagonist	RIG-I, MDA	WNV	BiFC, co-IP, IFA	Zhang et al. (2017) [85]
	MAVS	JEV	ChIP	Zhou et al. (2020) [86]
	CDK1	JEV	Co-IP	Li et al. (2021) [88]
	TBK1	ZIKV	Co-IP	Wu et al. (2017) [87]
	USP8	ZIKV	Co-IP	Zheng et al. (2018) [91]
Complement-mediated neutralization suppression	C4b	WNV, YFV, DENV	Co-IP, ELISA	Avirutnan et al. (2011) [89,90] Avirutnan et al. (2010)
Autophagy	AMPK	DENV	Co-IP, IFA	Wu et al. (2023) [92]
Cytoskeleton	NEK2, TAOK1	DENV	Co-IP, IFA	Dechtawewat et al. (2016) * [93]
	SUN2	ZIKV, DENV, JEV	Co-IP	Huang et al. (2024) [81]
Golgi interaction	COG1	DENV	Co-IP, IFA	Dechtawewat et al. (2016) * [93]
Viral RNA translation	CCT complex, OST complex	DENV	Co-IP	Hafirassou et al. (2017) * [59]
	RPL-18	DENV	Co-IP	Cervantes-Salazar et al. (2015) * [79]
Viral RNA replication	OS9, HERPUD1, HRD1, DERLIN1	JEV	IFA	Sarkar et al. (2024) [94]
	RACK1, SEL1L	DENV	Co-IP	Hafirassou et al. (2017) * [59]
RO stability	Vimentin	JEV, WNV ZIKV	Co-IP, IFA 3D-SIM, IFA	Xie et al. (2024) [80,95] Zhang et al. (2022)

* NS1-specific interactome study. IFA: immunofluorescence assay; Co-IP: co-immunoprecipitation; BiFC: bimolecular fluorescence complementation; ChIP: chromatin immunoprecipitation; ELISA: enzyme-linked immunosorbent assay; 3D-SIM: three-dimensional structured illumination microscopy.

### 3.2. Non-Structural Protein 2A

Non-structural protein 2A [NS2A; 226 a.a. (ZIKV)] is a multi-pass transmembrane protein that has confirmed roles in viral RNA synthesis, aiding in RO formation or stabilization, assembly, and a postulated role in viral RNA transport [96,97,98,99,100]. This hydrophobic protein is cleaved at the junction between NS1-NS2A by an unknown host protease acting within the ER lumen and at the C-terminal NS2A-NS2B junction by NS2B-3 (Figure 1C). The number of transmembrane domains (TMDs) within NS2A is debated, as one study suggests only a single pass, while many others report up to five [96,97]. The atomic structure of NS2A remains unsolved, and membrane topographical and mutagenesis studies have only been performed for mosquito-borne viruses, including DENV, ZIKV, and YFV [96,97,101]. NS2A has no inherent enzymatic activity and has been proposed as a scaffold for RO biogenesis by inducing negative membrane curvature [102]. A functional role of NS2A has been proposed during assembly by mediating the cleavage of structural proteins, transporting nascent viral RNAs to assembly sites, and assisting in nucleocapsid formation and incorporation [96,102]. The specific mechanisms of these proposed functions have not been deciphered primarily because the exact nature of virion assembly remains unresolved.

NS2A also functions to evade the innate immune system of the host by modulating interferon signaling and production in various flaviviruses (Table 2). In JEV, NS2A represses the cellular function of PKR, an antiviral host factor that reduces virus replication and translation [103]. PKR is induced by interferon and activated by dsRNA to phosphorylate the eukaryotic translation initiation factor 2α subunit (eIF2α), resulting in global translation arrest. In ZIKV, NS2A has been shown to block interferon production by suppressing RIG-I, IRF3, and other MDA5-induced downstream factors and the JAK-STAT signaling cascade by promoting the degradation of STAT1 and STAT2 [104,105,106]. Another aspect of the multifaceted roles NS2A plays within the cell is targeting host homeostatic mechanisms. Cells utilize autophagy of specific organelles, such as sections of the ER, termed ER-phagy, to maintain homeostasis and turnover of cellular materials. Flaviviruses hijack this phenomenon to ensure survival and prolong the infection. In ZIKV, NS2A, ubiquitinated by AMFR, has been shown to bind to and localize FAM134B, an ER-phagy receptor, to the proteasome for degradation, inhibiting the ER-phagy process and allowing the infection cycle to be sustained [107]. Validated host factor interactions with NS2A are shown below (Table 2).

### 3.3. Non-Structural Protein 2B

Non-structural protein 2B [NS2B; 130 a.a. (ZIKV)] is a hydrophobic protein embedded in the ER membrane following translocation and is the cofactor for NS3 protease function. After being cleaved in cis from NS3, NS2B is released from NS2A by an additional cleavage event by the newly formed NS2B-3 complex (Figure 1C). NS2B is predicted to have two to four transmembrane helices separated by a 47-amino-acid cytoplasmic linker interacting with NS3 to anchor it to the membrane and contribute to its substrate specificity [114,115,116,117,118]. In JEV and DENV, the transmembrane domains of NS2B were shown to interact with NS2A and serve essential roles in RNA synthesis. Mutations within this NS2A-interacting domain reduced viral replication and inhibited virion assembly, suggesting a synergistic role of NS2B with NS2A and potentially other non-structural proteins participating in the RO [116,119]. In DENV, NS2B was found to promote the lysosomal degradation of the cytosolic DNA sensor cGAS, inhibiting type-I interferon production and reducing activation of downstream ISGs (Table 3) [120]. A part of the cellular antiviral response is the detection of mitochondrial DNA and damage during infection through activation of the cGAS/cGAMP/STING pathway. An additional protein-protein interaction identified for NS2B is the nuclear envelope-localized host factor POM121C, potentially affecting nuclear pore stability [121]. NS2B-host factor validated interactions are summarized below (Table 3).

### 3.4. Non-Structural Protein 3

Non-structural protein 3 [NS3; 617 a.a. (ZIKV)] is a soluble protein that contains a serine protease domain and an NTPase-dependent helicase domain. The solubility of this protein is dependent upon the aid of NS2B, provided either in cis or trans, to properly fold as NS3 protease is non-functional *in vitro* without NS2B [123,124]. The monomeric crystal structure has been characterized in DENV, ZIKV, TBEV, WNV, JEV, and YFV (Figure 1E) [44,125,126,127,128,129]. The N-terminal region contains the chymotrypsin-like serine protease domain, and the C-terminal region consists of the NTPase and helicase domains connected by a 10-amino-acid flexible linker with a regulatory role [123,130]. Functionally, these two domains act independently, but the helicase domain may enhance protease activity, reminiscent of NS3 in HCV, a member of the *Hepacivirus* genus [123,125,130].

The protease domain, with the NS2B cofactor, herein referenced as NS2B-3, can process the viral polyprotein in both cis and trans on the cytosolic side of the ER membrane (Figure 1C). The substrate preferences of this domain, enhanced by NS2B, are typically dibasic or polybasic residues like arginine and lysine found in the recognition sequence [44]. The binding pocket of NS2B-3 displays the classical serine–protease catalytic triad (His51, Asp75, and Ser135) in the central cleft where the protease domain adopts a two β-barrel conformation with the catalytic triad positioned in the middle (Figure 1E) [44,125]. The C-terminal NTPase and helicase domains function during viral RNA synthesis to cap the nascent positive-sense RNAs and unwind the dsRNA intermediate, respectively. Belonging to the superfamily 2 (SP2) helicases, this domain displays α/β RecA-like folds with the NTP-binding P-loop constituting the ATP binding site and the RNA binding tunnel located downstream [44,126]. During the capping process, NS3 removes the 5′ terminal phosphate from the nascent positive-sense RNA, which allows NS5 to catalyze the addition of a guanosine monophosphate (GMP). The helicase domain participates in viral RNA synthesis through presumed direct interactions with the RdRp, likely following negative-strand synthesis to unwind the dsRNA intermediate and to begin nascent positive-strand synthesis. However, the helicase properties of NS3 may also function during the initial negative-strand synthesis step to break the secondary and tertiary structures of the genomic RNA template, indicating the critical synergistic effect of NS3 and NS5 cooperation. Beyond direct interactions with NS5, NS3 is known to rely on the cytosolic loops of NS4A and NS4B to remain within the RO. NS4B specifically assists NS3 in dissociating from single-stranded RNA (ssRNA), and mutations within both transmembrane proteins render the virus defective in replication [131]. Outside of enzymatic functions, NS3 is also postulated to have non-enzymatic roles in assembly [132,133]. These assembly-related functions are still under investigation and likely are synergized with other host or viral proteins.

NS3 and the NS2B-3 complex co-opt many host factor interactions to enable prolific virus replication and survival (Table 4). In an ectopic expression study, the DENV NS2B-3 complex interacted with flippases (ABCA4/7) and the CTLH E3 ubiquitin ligase complex (RANBP8, MAEA, WDR26) [121]. These interactors were not validated, so their direct role with NS3 is unknown. NS3 itself was described to interact with spindle assembly factors, ABCA4/7, and important trafficking factors (Sec61 and HOOK-Rab GTPase complex), facilitating vesicle movement along microtubules [121]. During DENV and ZIKV infection, the ERAD-related E3 ligases, HRD1 and RNF126, are known to modulate NS3 ubiquitination to impair its protease activity [134]. With regards to lipid biogenesis, exogenously expressed DENV NS3 was shown to localize with fatty acid synthase (FASN) and a *de novo* fatty acid biosynthesis enzyme (ACACA) and, in the context of infection, redistribute FASN to ROs to aid in their establishment and maintenance [135]. The protease complex also antagonizes the innate immune system of the host by impeding the JAK-STAT signaling pathway via JAK1 degradation [87]. Subsequently, this leads to limited expression of downstream ISGs and interferon production shutoff. Validated NS3-host interactions are summarized below (Table 4).

### 3.5. Non-Structural Protein 4A

Non-structural protein 4A [NS4A; 127 a.a. (ZIKV)] is a multi-pass transmembrane protein with four predicted TMDs (pTMDs) experimentally described but may contain up to seven alpha helices (Figure 1C). Biochemical analyses have shown that the N-terminal cytosolic region contains two amphipathic helices separated by an unstructured (or alpha-helical) linker that contributes to the homo-oligomerization present within ROs and has a high membrane affinity [34,141]. The second pTMD is predicted not to span the entire ER membrane and is inserted peripherally into the luminal leaflet due to being weakly hydrophobic to act as a wedge to remodel membranes (Figure 1C) [141,142]. The C-terminal helix, or the 2K peptide, is cleaved from NS4A by NS2B-3 and serves as a signal peptide sequence for proper NS4B topology. Occasionally, studies do not incorporate the 2K peptide as a constituent of NS4A, resolving the secondary structure consisting of only three pTMDs.

As a multi-pass transmembrane protein, the topology of NS4A relies on the endoplasmic reticulum membrane protein complex (EMC) to ensure proper orientation and integration [143,144]. Individual pTMDs can target and associate with membranes alone and will support viral replication in the context of a mature protein; however, different cleavage species across flaviviruses have separate roles. In DENV, removal of the NS4A-2K cleavage event impairs the membrane remodeling function, while this cleavage event is not required in WNV to effectively remodel ER membranes [34,141]. The NS4A-2K-NS4B precursor is known to interact with NS1 [75,145]. NS4A oligomerizes as a scaffold to curve the ER membrane into ROs and orchestrates a platform for NS3 and NS5 to be situated within the RO [146,147]. This NS4A scaffold may incorporate NS4B, NS1, and host factors to assist membrane bending and viral RNA replication [145]. Mutations within the N-terminus of NS4A from WNV, DENV, and ZIKV displayed defects in viral RNA synthesis, homo-oligomerization, and membrane remodeling due to their conserved nature across flaviviruses and indicate the reliance on this region to ensure the stability of the mature protein. The N-terminal region of WNV and JEV NS4A contains a cholesterol recognition amino acid consensus (CRAC) motif postulated to play essential roles in cytoplasmic membrane remodeling and RO biogenesis [148]. Whether or not this motif binds directly to cholesterol within these ER microdomains has yet to be shown. This region has also been demonstrated to assist in the NTPase, but not protease, activity of NS3, potentially acting as a cofactor in WNV and ZIKV [149].

NS4A interacts with host factors involved in membrane modification, RNA synthesis, RO anchoring, and antagonizing the innate immune response of the host to efficiently replicate the genome (Table 5). Cellular scaffolding and structural components like reticulon, vimentin, and others have been characterized as mediating interactions with NS4A from various flaviviruses to aid in the biogenesis and stability of the replication organelles. WNV, DENV, and ZIKV NS4A interact with reticulon (RTN3.1A) to assist in membrane remodeling and bending the replication organelle [150]. Vimentin was shown as a DENV NS4A interactor to prevent ROs from diffusing beyond the perinuclear region and was facilitated directly by the first 50 amino acids of NS4A [151]. The transmembrane protein 41B (TMEM41B) and vacuole membrane protein 1 (VMP1) have been demonstrated as pan-flavivirus interactors, where TMEM41B was shown to be recruited to ROs and interact with ZIKV NS4A potentially acting to assist in the membrane remodeling process [76,152]. The TMEM41B interaction with ROs may be further facilitated by VMP1, which can quickly diffuse between cell organelles, nearby vesicles, and lipid droplets, all of which are known to be co-opted during flavivirus replication and assembly [152,153]. ANKLE2, a scaffolding protein shown to mediate protein-protein interactions at the ER and inner nuclear membranes, has been identified to interact with NS4A within the RO, contributing to the induction of microcephaly in ZIKV [154]. NS4A has also been shown to interact with host factors to assist virus replication. An interaction with polypyrimidine tract-binding protein (PTB), which typically binds to the structured UTRs of the flavivirus genome, with NS4A was shown to promote negative-strand synthesis as the perturbation of this interaction pair reduced DENV replication [155]. NS4A is also known to activate specific IFN-I pathways to restrict viral replication.

The antiviral efforts of NS4A to suppress viral replication are known to be beneficial in fetal neural cells during ZIKV infection to prolong infection (Table 5). Exogenous expression of NS4A induced the upregulation of ISGs through activating the ISGF3 signaling pathway [156]. On the other hand, NS4A is known to inhibit the activity of the RLR-, but not TLR-, signaling pathway acting as a negative interactor. ZIKV NS4A outcompeted RIG-I and MDA5 binding to MAVS through the CARD domain, which decreases the interaction with its downstream effectors, TRAF6 and TBK1 [157,158]. Additionally, ZIKV NS4A was found to interact with and inhibit RIG-I/MDA5 signaling pathways factors except for IKKε and IRF3-5D [104,105]. In JEV, NS4A reduces the phosphorylation event of STAT1 and STAT2 by directly interacting with the helicase DDX42. This ATP-dependent helicase is a member of the DExD/H-box family of helicases to which RIG-I and MDA5 are members, so DDX42 may act as a cellular dsRNA sensor and NS4A-mediated inhibition of this helicase prevents downstream activation of the JAK-STAT pathway [159]. Beyond directly antagonizing the innate immune system of the host to suppress IFN production and subsequent activation in neighboring cells, flaviviruses also co-opt autophagy to remain hidden from cytosolic sensors (see Section 3.2).

Autophagy, from a flavivirus standpoint, is advantageously activated by the phosphoinositide 3-kinase (PI3K) function (Table 5). DENV NS4A was shown to upregulate PI3K-dependent induction of autophagy to prolong infection [160]. Also associated with DENV NS4A, the autophagy-related factors mTOR, CISD2, and PSEN1 were shown to interact when exogenously expressed. However, characterization of the interaction, whether positive or negative, was not pursued by the authors, leaving questions about the biological relevance of these host factors during flavivirus infection [121]. In human fetal neural stem cells (fNSCs), ZIKV NS4A, in cooperation with NS4B, promoted autophagy by inhibiting the Akt-mTOR signaling pathway by suppressing mTOR phosphorylation. Under the same conditions, this effect was not seen in DENV NS4A and NS4B counterparts, indicating a unique attribute with ZIKV infection in these neural cells [161]. NS4A also co-opts many other interactions with host factors related to mitochondrial targeting and organization, trafficking, and lipid biosynthesis, to name a few.

DENV NS4A was shown to bind and interact with protein complexes in the mitochondrial membrane, notably TOMM70A and TIMM44, and other mitochondrial-associated factors that participate in targeting or organization, such as SAMM50, CHCHD6, and MTX2 [121]. Additionally, DENV NS4A displayed a potential interaction with AGPAT5 and MBOAT7, both necessary acyltransferases related to lipid biosynthetic pathways [121]. Validated NS4A-host factor interactions are summarized below (Table 5).

**Table 5:** Summary of replication- and assembly-related NS4A protein interactors.

Virus-Adapted Function	Host Factor	Flavivirus	Validation Method	Reference
Innate immunity antagonization	DDX42	JEV	Co-IP, IFA	Lin et al. (2008) [159]
	MAVS	ZIKV	Co-IP, IFA, M2H	Ma et al. (2018) [157,158] Hu et al. (2019)
Autophagy	LC3	ZIKV	IFA	Liang et al. (2016) [161,162] Lee and Shin. (2023)
Mitochondria	PINK1	JEV	Co-IP, IFA, Y2H, PLA	Agarwal et al. (2022) * [163]
Viral RNA translation	STT3A/B	DENV	Co-IP	Marceau et al. (2016) [113]
Viral RNA replication	PTB	DENV	Co-IP, IFA, Y2F	Jiang et al. (2009) [155]
Membrane perturbation	Reticulon	DENV, WNV, ZIKV	Co-IP, IFA, FRET	Aktepe et al. (2017) [150]
	Vimentin	DENV	Co-IP, IFA, PLA, SEM	Teo and Chu. (2014) * [151]
	TMEM41B VMP1	All flaviviruses	Co-IP, IFA	Hoffmann et al. (2021) [152]
	ANKLE2	ZIKV	IFA	Fishburn et al. (2025) [154]

* NS4A-specific interactome study. IFA: immunofluorescence assay; Co-IP: co-immunoprecipitation; PLA: proximity-ligation assay; M2H: mammalian-two hybrid; Y2H: yeast-two hybrid; FRET: fluorescence resonance energy transfer; SEM: scanning electron microscopy.

### 3.6. Non-Structural Protein 4B

Non-structural protein 4B [NS4B; 251 a.a. (ZIKV)] is a multi-pass transmembrane protein, like NS2A, NS2B, and NS4A. Mature NS4B proteins are generated following the dual-cleavage action of host SPase and NS2B-3 at the 2K-NS4B and NS4B-NS5 junctions, respectively (Figure 1C). Experimentally, establishing the membrane topology of NS4B has been challenging as many studies differ in their conclusions across the various flaviviruses, but it is predicted to contain nine alpha helices [141,164,165,166,167,168]. Most studies agree that NS4B contains at least five pTMDs that are hydrophobic enough to insert into or associate with the ER membrane. However, which pTMDs are integrated through the membrane or peripherally associated is debated (Figure 1C) [144]. Interestingly, pTMD5, the most C-terminal pTMD, may diffuse from the cytoplasmic side of the ER membrane to the ER lumen following cleavage at the NS4B-NS5 junction [166,167]. Following membrane integration, DENV and WNV pTMD4 and pTMD5, along with the cytoplasmic loop, were determined to be important in mediating the dimerization of NS4B [131,169].

NS4B participates in viral RNA replication by acting as a scaffold for the RO, in conjunction with NS4A, host factors, and potentially NS1 dimers [131,169,170] (see Section 3.5). When overexpressed, WNV NS4B was sufficient to induce these compartments within the ER membrane [171]. Mutations within DENV NS4B are known to suppress viral RNA synthesis, likely due to impaired dimerization [131,169]. The physical interaction between the cytosolic loop of NS4B and NS3 negatively contributes to the helicase function of NS3, allowing it to dissociate from ssRNA [170,172] (Section 3.4). This interaction has gained interest as a potentially potent antiviral target using the small-molecule inhibitors JNJ-A07, JNJ-1802, and NITD-688 against DENV [173,174,175]. JNJ-1802 (Mosnodenvir; NCT05201794) and NITD-688 (EYU688; NCT06006559) were selected to proceed into clinical trials. However, Mosnodenvir has been discontinued after phase 2a, but EYU688 remains active. Like NS1, NS4B has also been shown to potentially undergo OST-mediated N-linked glycosylation events that may contribute to viral RNA synthesis [59,166,176].

Akin to other flavivirus non-structural proteins mentioned elsewhere, NS4B participates in antagonizing the innate immune system of the host (Table 6). Full-length NS4B of WNV, JEV, YFV, and ZIKV, as well as the N-terminal region of DENV NS4B, are sufficient for blocking STAT1 and/or STAT2 phosphorylation and suppressing TBK1-mediated IFN-α/β signaling [87,177,178]. The cooperation of NS4A and NS4B has been shown to affect the Akt-mTOR phosphorylation activity, leading to increased cellular dysregulation and viral persistence in ZIKV [161]. Related to this cellular death pathway, ZIKV NS4B was identified localizing near the mitochondrial outer membranes (OMM), otherwise known as a mitochondrial-associated membrane (MAM), activating Bax, a pro-apoptotic host factor, to induce mitochondrion-mediated apoptosis within neural progenitor cells potentially contributing to microcephaly [179]. DENV NS4B localizing to these regions’ neighboring mitochondria has also been shown to reduce RIG-I-mediated signaling by inhibiting a critical mitochondrial fission factor, DRP1 [180]. JEV and DENV NS4B proteins were found to directly interact with the (VCP)/p97-NPL4 complex to sequester it away from PKR to inhibit downstream IFN activation [181,182]. A summary of validated NS4B-host factor interactions is shown below (Table 6).

### 3.7. Non-Structural Protein 5

Non-structural protein 5 [NS5; 903 a.a. (ZIKV)] is a highly conserved protein comprising an S-adenosylmethionine (SAM) methyltransferase (MTase) domain, responsible for methylating the nascent viral RNA, a nuclear localization signal (NLS), and an RNA-dependent RNA polymerase (RdRp) domain, which replicates the RNA genome of the virus (Figure 1F). While NS5 has no intrinsic membrane association, it is recruited to the viral replication complex by interacting with NS3 [185]. Full-length structures of NS5 have been solved for ZIKV, YFV, DENV, and JEV (Figure 1F) [186,187,188,189], whereas the MTase and RdRp domains have been characterized independently for WNV and TBEV [190,191,192].

The N-terminal MTase domain of NS5 contains a conserved α/β/α sandwich structure, consistent with SAM-dependent MTases, with a conserved Lys-Asp-Lys-Glu catalytic tetrad [192]. The MTase domain binds GTP, converting it to a GMP-enzyme intermediate, adding the guanosine to a 5′ diphosphate on the nascent RNA [193]. The MTase domain then catalyzes sequential methylation of GpppA-RNA at the N-7 and 2′-O sites, resulting in the m7GpppAm-RNA cap [192]. Loss of methylation at the 2′-O site attenuates the virus, whereas loss of methylation at the N-7 site completely abolishes viral replication [192]. The linker region between the MTase and RdRp domains acts as a nuclear localization sequence, translocating NS5 to the nucleus when not maintained within a replication organelle [194].

The RdRp domain is highly conserved among flaviviruses and is similar to many other viral RdRps, consisting of finger, palm, and thumb subdomains [195]. The RdRp synthesizes the negative-strand RNA by first interacting with the 5′ SLA and 3′ SL, then initiating *de novo* synthesis of RNA from the 3′ end through the priming loop, which enables the addition of a dinucleotide primer specifically recognizing the 3′ conserved CU sequence in flaviviruses. Similarly, the 3′ CU sequence in the negative strand (5′ AG in the positive strand) acts as the initiation sequence for nascent positive-strand synthesis. The reliance of the flavivirus RdRp on this dinucleotide sequence is due to the ability of the priming loop to maintain a dinucleotide primer for initiation, leading to extremely high conservation of the 5′ AG and 3′ CU motifs in flavivirus genomes [196].

Post-translational modifications to NS5 by the host modulate the activity and localization of NS5, influencing its role in replication. Phosphorylation of YFV and DENV NS5 at Ser56 by an unknown host kinase occurs in vivo and prevents 2′-O methylation by the MTase domain of NS5 [197]. Phosphorylation of DENV NS5 by the mosquito kinase PKG was shown to occur *in vitro*, and loss of the phosphorylated residue resulted in loss of replication [198]. Pryor et al. (2007) showed an enrichment of hyperphosphorylated NS5 in nuclear fractions, indicating a role of phosphorylation in the nuclear translocation of NS5 [199]. DENV NS5 is SUMOylated in vivo, dependent on the host factor Ubc9, a SUMO conjugase. It is theorized to stabilize NS5 against degradation, as significantly lower levels of NS5 are detected if SUMOylation is lost [200]. Ubiquitination of YFV NS5 in an IFN-α/β-dependent manner mediates interaction with STAT2, promoting viral shutdown of STAT2 [201].

NS5 interacts with several host factors to antagonize the host antiviral response, promoting efficient viral replication (Table 7). One essential interaction for flaviviruses is the prevention of type-I IFN signaling. In DENV infection, NS5 interacts with human STAT2 (hSTAT2) and UBR4 to promote the degradation of hSTAT2, preventing type-I IFN activation. YFV similarly degrades hSTAT2, requiring IFN activation to occur first, which leads to the ubiquitination of NS5, strengthening its association with hSTAT2 [201]. Shah et al. (2018) demonstrated the inhibition of PAF1C recruitment by NS5 to inhibit ISG transcription [121]. DENV NS5 depends on the host factor Hsp70 to properly fold and stabilize [202]. Further virus-host interactions for DENV NS5 were identified by Poyomtip et al. (2016) using a live recombinant DENV with an His/FLAG affinity-tagged NS5. Poyomtip et al. (2016) identified HSPA5, ENO1, TRIM21, and FGA, previously known interactors of the DENV NS5 protein. Additionally, they identified 93 other host proteins with high confidence, which clustered into translational regulation, membrane-bound vesicles, mRNA splicing, ribonucleoprotein complexes, and cytosolic proteins. Twelve of these hits were also identified as being differentially expressed during infection [203]. Many of these hits have yet to be characterized for relevance to the viral life cycle but provide promising leads for future study of NS5 host interactions. A summarized list of validated NS5-host interactions is shown below (Table 7).

## 4. Virus-Host Interactions

### 4.1. Large-Scale Screenings for Virus-Host Interactions

Advances in large-scale proteomic and genetic methods have improved our ability to identify interactomes between hosts and viruses, significantly advancing our understanding of the complex interactions between flaviviruses and their hosts. Specifically, advances in CRISPR and RNAi libraries have enabled rapid whole-genome screens for interactors, and advances in tagging methods and proteomics data processing have improved the workflow for affinity-purification mass-spectrometry (AP-MS) and proximity labeling, making it easier than ever to construct interactomes for viruses. This section will focus on the application of these cutting-edge techniques to flaviviruses and the resulting findings.

#### 4.1.1. Genetic-Based Screening Methods

RNAi, ectopic overexpression, and CRISPR-based methods are well-established in identifying host factors, with improvements in validated pre-constructed libraries promising to enhance their applicability [210]. These methods have been employed to identify virus-host interactions in several flaviviruses [211,212,213,214]. While genetic screening methods excel in discovering direct and indirect interactions, distinguishing between the two is challenging and requires downstream validation. RNAi allows genome-wide knockdowns and is applicable in numerous cell lines and organisms but struggles with variable knockdown efficiency across genes. Ectopic overexpression is an approachable, rapid method for researchers to induce the gain-of-function of potential antiviral or proviral host factors. However, this method requires efficiently transfected or transduced cells and may result in false hits due to significant variations from endogenous expression. CRISPR methods offer precise control of gene expression, like knockout, reduced, or increased expression, but struggle with heterozygous phenotypes in diploid cells or require haploid cells, limiting applicability [215].

Lesage et al. (2022) used RNAi to identify interferon-stimulated genes (ISGs) impacting ZIKV replication. For this study, 386 ISGs were knocked down in microglial cells stimulated with IFNα2 then infected with ZIKV, identifying nine antiviral and 12 proviral genes [211]. The enriched genes were further characterized against DENV and WNV. Several previously unknown proviral and antiviral factors were discovered, and ones previously characterized were validated. The authors selected ISGs based on previous work in primary T-cells activated by monocytes rather than ZIKV-targeted cells, potentially missing relevant interactions. Other methods adapted RNAi to high-throughput screens, allowing full genome screening to rapidly identify hits [216]. Barrows et al. (2019) implemented a library targeting nearly 23,000 mRNAs in Huh7 cells to determine DENV essential host factors. Combined with a previous YFV screen, 274 common proviral factors clustering around ribosomal subunit proteins and ER translation factors were identified. The authors validated multiple subunits of the ER membrane protein complex (EMC) as potent proviral proteins for DENV and YFV but not WNV. This study highlights the value of large-scale RNAi screens in identifying unique proviral factors [217]. RNAi thus provides a powerful tool for identifying host factors necessary for viral replication but may include false positives and negatives due to off-target silencing and non-specific suppression of viruses due to immune system activation [218]. Genome-wide ectopic overexpression by Petrova et al. (2018) established essential factors for YFV and WNV in non-permissive cells. The authors used a shotgun library screen from A549 cells, covering most coding DNA and identifying 17 proviral host factors. Subsequently, selected hits, RPL19, IPO9, and DDOST, were validated via RNAi, of which DDOST promoted efficient RNA replication, and RPL19 promoted translation [213].

CRISPR methods are increasingly used to probe virus-host interactions due to the development of high-throughput libraries coupled with next-generation sequencing (NGS) to identify hits. Li et al. (2019) used a CRISPR library screen to identify factors leading to the resistance of ZIKV infection in human neuronal cells. A library of sgRNAs targeting 18,663 genes was used in induced pluripotent stem cell-derived neural progenitor cells (iPSC-derived NPCs) to identify genes that conferred increased cellular survival when knocked out. The authors confirmed hits via targeted CRISPR-KO and pharmacological suppression, demonstrating proviral activity for EMC2, SSR2, SSR3, and ISG15 [214]. CRISPR activation (CRISPRa) upregulates expression levels with high-throughput capabilities [215]. Luu et al. (2021) used a genome-wide CRISPRa screen to identify RhoV and WWTR1 as proviral ZIKV factors in IFN-deficient human fibroblasts, enabling the identification of new interactions previously overlooked in ectopic overexpression and loss-of-function screens [219].

#### 4.1.2. Protein-Based Screening Methods

Protein-based methods interrogate interactions between virus and host proteins more directly than genetic methods. Wide application of AP-MS, proximity labeling, and yeast two-hybrid (Y2H) methods have provided invaluable information about the interactomes of individual flavivirus proteins with the host. Scaturro et al. (2018) used AP-MS to establish interactomes for all ZIKV proteins, except NS2A, in SK-N-BE2 cells, identifying 386 high-confidence interactors and further validating six host-dependency factors [76]. Coyaud et al. (2018) combined AP-MS and BioID to generate interactomes for all ZIKV proteins, identifying 1224 interactors and shedding light on a range of cellular pathways essential for ZIKV replication [220].

In the application of AP-MS, the target protein is often ectopically expressed with a purification tag, such as employed by Scaturro et al. (2018) [76]. While ectopic expression of the target protein can be effective, it can miss interactions that require the presence of other viral proteins or complexes constructed only in the context of active infection. For this reason, inserting tags into a full-length virus can be beneficial, as performed by Maio et al. (2016). A dual-strep tag was added to the DENV NS5, identifying and characterizing interactions with the cellular spliceosome [208]. A similar replication-competent DENV with affinity-tagged NS5 was generated by Poyomtip et al. (2016) [203] and had findings consistent with Maio et al. (2016) [208]. Chatel-Chaix et al. (2015) also performed AP-MS from infected cells using an HA-tagged ZIKV NS4B, characterizing interactions with other ZIKV proteins [170]. Introducing peptide tags into a live virus maintains a context relevant to infection for the cellular proteome, enabling more accurate and reliable identification of hits. While AP-MS provides a powerful tool for identifying interacting proteins, it often suffers from bias towards high-affinity interactions, as transient and weak interactors are frequently lost during purification.

Proximity labeling methods have been employed in flaviviruses to elucidate interactomes, filling some gaps missed in AP-MS studies [220]. Coyaud (2018) and coworkers generated an interactome of each ZIKV protein using both AP-MS and BioID, providing a strong comparison of the two methodologies. AP-MS was able to identify more cytosolic interactors but failed to identify many membrane proteins, whereas BioID demonstrated an opposite bias. In the study, an overlap of 165 hits between the two data sets and 523 (BioID) and 536 (AP-MS) total unique hits were identified [220]. Tang et al. (2023) performed TurboID on the TBEV NS2A by stably expressing a TurboID-NS2A fusion and infecting cells with wild-type TBEV. This study identified immune response factors spatially near NS2A and 175 other interactions differentially altered during infection [111]. While proximity tags fail to distinguish between direct and spurious interactions due to compartmentalization, they provide an orthogonal approach to AP-MS, enabling the identification of membrane-bound proteins and transient interactors more readily.

Y2H was performed by Golubeva et al. (2020) in conjunction with AP-MS to generate an interactome for each of the ZIKV non-structural proteins. Golubeva et al. (2020) identified 109 interactors from a human brain cDNA library, specifically characterizing the stabilizing interaction of ZIKV NS5 with PIAS1, a SUMO ligase [221]. This interaction is consistent with previous findings for DENV NS5 being stabilized by SUMOylation [200]. The limitations of this methodology are that Y2H screens identify only interactions that occur readily between two proteins and struggle to identify membrane-bound proteins that do not localize to the nucleus or proteins that require cofactors and/or post-translational modifications to properly fold [222].

### 4.2. Summary of Large-Scale Screenings

The use of large-scale genetic and proteomic methods to identify flavivirus-host interaction networks has greatly advanced the understanding of the viral life cycle. Alone, each technique can identify different classes of interactions, but when combined, many more interactors can be elucidated. Often, genetic methods fail to identify interactors with functionally redundant homologs within a cell, but they can identify genes that affect viral replication and assembly without directly interacting with viral proteins. Conversely, proteomic methods excel at identifying interactors that have redundancy and building networks based on shared functions, elucidating interactions that are difficult to identify in genetic screens. Genetic screens have been successfully employed to build interaction networks for several flaviviruses, notably ZIKV, DENV, and YFV [211,213,217], and advances in specialized CRISPR techniques, such as CRISPRi and CRISPRa, may lead to further identification of hits that have been missed due to toxicity in complete knockout or extreme overexpression. Protein-based methods have been heavily applied to establishing interactomes in flaviviruses [76,121,220,221,223,224], but most methods have relied on expression constructs rather than live viruses. Further investigation of proteome interactions with functionally tagged live viruses may improve understanding of virus-host interactions in an active infection context. Many interactors from high-throughput screens have limited validation, and future studies may aim to establish the biological significance of these interactions. A comprehensive summary of studies using high-throughput methods to establish flavivirus interaction networks is shown below (Table 8).

## 5. Conclusions

Virus-host interactions are foundational to understanding the key aspects of the virus life cycle. Within flaviviruses, the host supplies the cellular materials to replicate the viral genome and generate viral proteins, as well as the physical structures the virus utilizes to hide from innate immune sensors and assemble the virion. Many methodologies exist to identify these interactions, including genetic approaches like CRISPR and RNAi screening and proteomic methods such as AP-MS and proximity labeling. Combining these techniques validates unique and enriched hits, providing the framework for deciphering the biological relevance and molecular basis of these interactions. Currently, ectopically expressed viral proteins and their proteomic spheres predominate the content of interactome studies. While these studies are highly beneficial in understanding the potential complexes or pathways that a specific viral protein may co-opt, the main component missing is the context of infection. As seen in the overviews of each non-structural protein covered in this review, the viral non-structural proteins (NS1–NS5) fulfill many roles during the viral life cycle and rarely act alone.

NS1, a soluble protein with dimer and hexamer structures, supports the biogenesis of the replication organelle by inducing positive curvature into the membrane and impairing host immune systems via vascular leakage and tissue-specific damage. NS2A is an integral membrane protein that functions within the replication organelle and may have chaperoning functions during assembly. NS2B mainly serves as the cofactor for the viral protease. NS3, another soluble viral protein, constitutes three main functions—helicase, protease, and NTPase—that act during the RNA synthesis and translation phases of the viral life cycle. NS4A is a transmembrane protein that oligomerizes to form the replication organelle and provides a scaffold for NS3 and NS5. NS4B functions similarly to NS4A with the additional interaction with NS1 to induce membrane curvature and potentially co-opt lipid synthetic pathways. NS5 is an RNA-dependent RNA polymerase synthesizing new viral genomes and capping them with MTase activity. These non-structural proteins co-opt host pathways to ensure efficient replication and assembly, including antagonizing the innate immune systems of the host, redirecting cellular machinery for *de novo* material production, and targeting certain host factors for degradation to prolong infection. Understanding these virus-host interactions is foundational in unraveling the complexities of the flavivirus life cycle.

### Future Work

It is critical to determine the biological relevance of the hits discovered and to identify why the virus may co-opt or inhibit a specific cellular function. Like viral proteins, interactions with the host are complex and multifaceted, so determining the sole function of virus-host interactions is likely impossible. Interactome studies are expansive and information-heavy, restricting the ability to pursue multiple interactions and lessening the scope of some very foundational work. However, these studies are incredibly impactful, and incorporating findings from interactomes will broaden the knowledge of the complex intertwining of cell biology and virology. In the future, interactome studies can be more impactful if enhanced by establishing interactomes in the context of active infection, warranting the simultaneous presence of viral factors and their interactors.

## Figures and Tables

**Table 2:** Summary of validated replication- and assembly-related NS2A protein interactors.

Virus-Adapted Function	Host Factor	Flavivirus	Validation Method	Reference
Innate immunity antagonist	PKR	JEV	Co-IP	Tu et al. (2012) [103]
	TRIM52	JEV	Co-IP, IFA	Fan et al. (2016) [108]
	STAT1/2	ZIKV	Co-IP, IFA	Fanunza et al. (2020) [106]
Autophagy	FAM134B	DENV, JEV, WNV, ZIKV	Co-IP, IFA	Zhang et al. (2024) [107]
Neuropathy	N-cadherin, ZO-1, β-catenin, SMAD7, NUMBL, ARPC3	ZIKV	Co-IP, IFA, Microarray	Yoon et al. (2017) * [109]
	LAMP2A, KPNA2	ZIKV	Co-IP	He et al. (2020) [110]
Viral assembly	MST3	TBEV	Co-IP, IFA	Tang et al. (2023) * [111]
Membrane perturbation	ADAM15	TBEV	Co-IP, IFA	Yang et al. (2021) [112]
Viral translation	STT3A/B	DENV	Co-IP	Marceau et al. (2016) [113]
Inflammasome activation (vascular leakage)	NLRP3	DENV	Co-localization	Shrivastava et al. (2020) [98]

* NS2A-specific interactome study. IFA: immunofluorescence assay; Co-IP: co-immunoprecipitation.

**Table 3:** Summary of validated replication- and assembly-related NS2B protein interactors.

Virus-Adapted Function	Host Factor	Flavivirus	Validation Method	Reference
Innate immunity antagonist	cGAS	DENV	Co-IP, IFA	Aguirre et al. (2017) [120]
Viral RNA translation	STT3A/B	DENV	Co-IP	Marceau et al. (2016) [113]
	SPCS1	JEV, WNV, ZIKV	BiFC, Co-IP	Ma et al. (2018) [122]

IFA: immunofluorescence assay; Co-IP: co-immunoprecipitation; BiFC: bimolecular fluorescence complementation.

**Table 4:** Summary of replication- and assembly-related NS3 protein interactors.

Virus-Adapted Function	Host Factor	Flavivirus	Validation Method	Reference
Innate immunity antagonization	JAK1	ZIKV	Co-IP	Wu et al. (2017) [87]
Particle assembly	CHMP4A	TBEV	Co-IP, IFA	Tran et al. (2022) [136]
	TSG101	JEV	Co-IP, IFA, TEM	Chiou et al. (2003) [137]
Viral RNA translation	STT3A/B	DENV	Co-IP	Marceau et al. (2016) [113]
	HRD1	JEV, DENV	Co-IP, IFA	Ci et al. (2023) [92,134] Wu et al. (2024)
	RNF126	DENV, ZIKV	Co-IP, IFA	Ci et al. (2023) [134]
Viral RNA replication	KAT5γ	DENV, WNV, YFV, ZIKV	Co-IP, PLA	Serman et al. (2023) [138]
	ALIX	TBEV, YFV	Co-IP, IFA, ELISA	Tran et al. (2022) [136,139] Carpp et al. (2011)
RO biogenesis	FASN	DENV	Co-IP, co-localization, Y2H	Heaton et al. (2010) [135]
	Rab18	DENV	Co-IP, co-localization	Tang et al. (2014) [140]

IFA: immunofluorescence assay; Co-IP: co-immunoprecipitation; Y2H: yeast-two hybrid; TEM: transmission electron microscopy; ELISA: enzyme-linked immunosorbent assay.

**Table 6:** Summary of replication- and assembly-related NS4B protein interactors.

Virus-Adapted Function	Host Factor	Flavivirus	Validation Method	Reference
Innate immunity antagonization	STAT1/2	DENV, WNV DENV, YFV	IFA, Luc, WBLuc, WB	Liu et al. (2005) [177,178] Muñoz-Jordán et al. (2005)
	TBK1	ZIKV	Co-IP, Luc	Wu et al. (2017) [87]
Mitochondria	Bax	ZIKV	Co-IP	Han et al. (2021) [179]
Membrane perturbation	TMEM41B	All flaviviruses	Co-IP, IFA	Hoffmann et al. (2021) [152,180] Chatel-Chaix et al. (2016) *
	ADAM15	TBEV	Co-IP, WB	Yang et al. (2021) [112]
	LNP	LGTV, WNV	Co-IP, IFA	Tran et al. (2021) [183]
	VCP	DENV DENV, JEV	Co-IP, IFA, WB Co-IP, WB	Mazeaud et al. (2021) [181,182] Arakawa et al. (2022)
	NPL4	DENV, JEV	Co-IP, IFA, WB	Arakawa et al. (2022) [182]
	CypA	YFV	IFA, Luc	Vidotto et al. (2017) * [184]

* NS4B-specific interactome study. IFA: immunofluorescence assay; Co-IP: co-immunoprecipitation; WB: western blot; Luc: luciferase reporter.

**Table 7:** Summary of replication- and assembly-related NS5 protein interactors.

Virus-Adapted Function	Host Factor	Flavivirus	Validation Method	Reference
Innate immunity antagonization	STAT2	ZIKV, DENV, WNV, YFV	IFA, WB, Luc	Grant et al. (2016) [204]
	PAF1C	ZIKV, DENV	Co-AP, comparative proteomics	Shah et al. (2018) [121]
		JEV	RNAi, Co-AP	Kovanich et al. (2019) [205]
Protein Folding and stabilization	Hsp70	DENV	RNAi, chemical inhibition	Taguwa et al. (2015) [202]
	UBC9	DENV	RNAi, Co-AP	Su et al. (2016) [200]
	HSPA5, DERL2	DENV	Co-AP	Mairiang et al. (2013) [206]
	PPP6C	ZIKV, JEV	Co-AP, AP-MS	Kovanich et al. (2019) [205]
Mitochondria	Ajuba	ZIKV	IFA, Co-AP	Ponia et al. (2021) [207]
Viral RNA replication	CD2BP2, DDX23, EFTUD2	DENV	WB, IFA	Maio et al. (2016) [208] *
	CyPA	WNV, DENV, YFV	Co-IP, RNAi, chemical inhibition	Qing et al. (2009) [209]

* NS5-specific interactome study. IFA: immunofluorescence assay; Co-IP: co-immunoprecipitation; Co-AP: co-affinity purification; WB: western blot; RNAi: RNA interference; Luc: luciferase reporter.

**Table 8:** Summary of flavivirus non-specific interactome studies.

Method	Cell Line	Virus	# of Hits	Validated Hits	Reference
CRISPR-KO	Huh7.5.1 HAP1	DENV	30 36	ERAD factors, OST complex	Marceau et al. (2016) [113]
CRISPR-KO	iPSC NPCs	ZIKV	130	EMC2, SSR2, SSR3, ISG15	Li et al. (2019) [214]
CRISPR-KO	Huh7.5.1 HEK293FT	DENV, WNV, ZIKV	5	EMC1-5	Ngo et al. (2019) [143]
CRISPR-KO	HAP1	DENV	3	Vigilin, SERBP1, ZNF598	Brugier et al. (2022) [225]
CRISPR-KO	HAP1	DENV	17	DPM1, DPM3	Labeau et al. (2020) [226]
CRISPR-KO	HAP1	ZIKV, YFV	?	TMEM41B, VMP1	Hoffmann et al. (2021) [152]
CRISPR-KO	Huh7.5	ZIKV	6	RACK1	Shue et al. (2021) [227]
CRISPR-KO	A549	WNV	?	OAS3, RNase L	Li et al. (2016) [228]
CRISPR-KO	Huh7.5	YFV, WNV, ZIKV	17	IFI6	Richardson et al. (2018) [229]
CRISPR-KO	HEK293FT	WNV	7	SEL1L, UBE2J1, EMC3, EMC2, DERL2, UBE2G2	Ma et al. (2015) [230]
CRISPRa	Human STAT1^−/−^ fibroblasts	ZIKV	2	RhoV, WWTR1	Luu et al. (2021) [219]
CRISPRa	Huh7	ZIKV	250	TMEM120A	Li et al. (2022) [231]
CRISPRa	Huh7	ZIKV	12	IFI6, IFNλ2	Dukhovny et al. (2019) [232]
RNAi	D.Mel-2	DENV	116	Nuclear Proteins	Sessions, et al. (2009) [233]
RNAi	HeLa	WNV	305	ERAD factors	Krishnan et al. (2008) [212]
RNAi	HMC3	DENV, ZIKV, WNV, YFV	386	APOL3, ISG15, LY6E, USP18, NAPA, MTA2	Lesage et al. (2022) [211]
RNAi	Huh 7	DENV, YFV, ZIKV	455	EMC1-5, TTC35, TMEM111	Barrows et al. (2019) [217]
RNAi + CRISPR KO	HeLa	DENV, ZIKV, YFV	150 (DENV) 11 (ZIKV)	EMC1-5, AXL	Savidis et al. (2016) [234]
AP-MS	HEK293T	WNV	26	PYM1	Li et al. (2020) [223]
AP-MS	HEK293T	DENV, ZIKV	28	PAF1C complex, SEC61, ANKLE2	Shah et al. (2018) [121]
AP-MS	SK-N-BE2	ZIKV	386	ATPase, VDACs, COX factors	Scaturro et al. (2018) [76]
AP-MS	HepG2	DENV	40	AUP1	Zhang et al. (2018) [224]
BioID + AP-MS	HEK293 T-Rex	ZIKV	688 (BioID) 701 (AP-MS)	OFD1, CEP85, PEX3, PEX19	Coyaud et al. (2018) [220]
Gain-of-function	HT1080	YFV, WNV	9	DDOST, RPL19, IPO9	Petrova et al. (2018) [213]
Gain-of-function	Human STAT1^−/−^ fibroblasts	YFV	5	IFI6, IFITM3	Schoggins et al. (2011) [235]
AP-MS + Y2H	HEK293FT	ZIKV	109 (Y2H) 89 (AP-MS)	PIAS1	Golubeva et al. (2020) [221]
Microarray	N/A	ZIKV, DENV	1708 (ZIKV) 1408 (DENV)	PSMC3, PSMA1, OVOL2, proteosome	Song et al. (2021) [236]
